# Smad4 represses the generation of memory-precursor effector T cells but is required for the differentiation of central memory T cells

**DOI:** 10.1038/cddis.2015.337

**Published:** 2015-11-19

**Authors:** J Cao, X Zhang, Q Wang, G Qiu, C Hou, J Wang, Q Cheng, Y Lan, H Han, H Shen, Y Zhang, X Yang, B Shen, J Zhang

**Affiliations:** 1 Department of Molecular Immunology, Institute of Basic Medical Sciences, Beijing, PR China; 2 Genetic Laboratory of Development and Diseases, State Key Laboratory of Proteomics, Institute of Biotechnology, 20 Dongdajie, Beijing, PR China; 3 Department of Medical Genetics and Developmental Biology, Fourth Military Medical University, Xi'an, PR China; 4 Shanghai Institute of Immunology, Institute of Medical Sciences, Shanghai Jiaotong University School of Medicine, Shanghai, PR China; 5 Key Laboratory of Medical Immunology, Ministry of Health, Peking University Health Science Center, Beijing, PR China

## Abstract

The transcriptional regulation underlying the differentiation of CD8^+^ effector and memory T cells remains elusive. Here, we show that 18-month-old mice lacking the transcription factor Smad4 (homolog 4 of mothers against decapentaplegic, *Drosophila*), a key intracellular signaling effector for the TGF-*β* superfamily, in T cells exhibited lower percentages of CD44^hi^CD8^+^ T cells. To explore the role of Smad4 in the activation/memory of CD8^+^ T cells, 6- to 8-week-old mice with or without Smad4 in T cells were challenged with *Listeria monocytogenes*. Smad4 deficiency did not affect antigen-specific CD8^+^ T-cell expansion but led to partially impaired cytotoxic function. Less short-lived effector T cells but more memory-precursor effector T cells were generated in the absence of Smad4. Despite that, Smad4 deficiency led to reduced memory CD8^+^ T-cell responses. Further exploration revealed that the generation of central memory T cells was impaired in the absence of Smad4 and the cells showed survival issue. In mechanism, Smad4 deficiency led to aberrant transcriptional programs in antigen-specific CD8^+^ T cells. These findings demonstrated an essential role of Smad4 in the control of effector and memory CD8^+^ T-cell responses to infection.

CD8^+^ cytotoxic T cells play pivotal roles in the clearance of intracellular pathogens.^1, 2^ Antigen-specific naive CD8^+^ T cells undergo a massive clonal expansion as they come in contact with their cognate antigen on activated antigen-presenting cells. Within the expanded clone, there exist distinct subsets that can be characterized by both function and phenotype. Cells expressing high levels of killer cell lectin-like receptor G1 (KLRG1) and low levels of IL-7 receptor-*α* (CD127) represent terminally differentiated, short-lived effector T cells (SLEC), whereas KLRG1^low^CD127^hi^ CD8^+^ T cells have a greater potential to enter into the memory pool.^3, 4^ In response to antigen restimulation, memory CD8^+^ T cells rapidly proliferate and differentiate into cytolytic T lymphocytes that confer enhanced protection against intracellular pathogens.

The transcriptional regulation of these cell-fate decisions has been an area of active research. It has been demonstrated that the T-box transcription factor T-bet (encoded by *Tbx21*) promotes CD8^+^ T-cell differentiation into short-lived effectors.^5, 6^ Eomesodermin (Eomes), B-cell CLL/lymphoma 6 (Bcl6), and T-cell factor 7 (Tcf7, also known as Tcf1, downstream transcription factor of the Wnt pathway) are required for important aspects of memory CD8^+^ T-cell generation.^5, 7, 8, 9^ In addition, B lymphocyte-induced maturation protein 1 (Blimp1, encoded by *Prdm1*) is essential for the differentiation of short-lived cytotoxic effector T cells and memory responses.^10, 11^ A recent study based on high-resolution microarray analyses has suggested that many other transcription factors are involved in these cell-fate decisions.^12^ Surprisingly, Smad4 (homolog 4 of mothers against decapentaplegic, *Drosophila*), a key intracellular signaling effector for the TGF-*β* superfamily, has been predicted as an activator.^12, 13^ Thus, it is of importance to identify the role of Smad4 in the differentiation of CD8^+^ effector and memory T cells. Here, we report that Smad4 is required for the differentiation of effector CD8^+^ T cells and memory responses.

## Results

### Eighteen-month-old *Smad4*^*co/co*^*;Lck-Cre* mice exhibit impaired CD44 expression in CD8^+^ T cells

**Figure 1:**
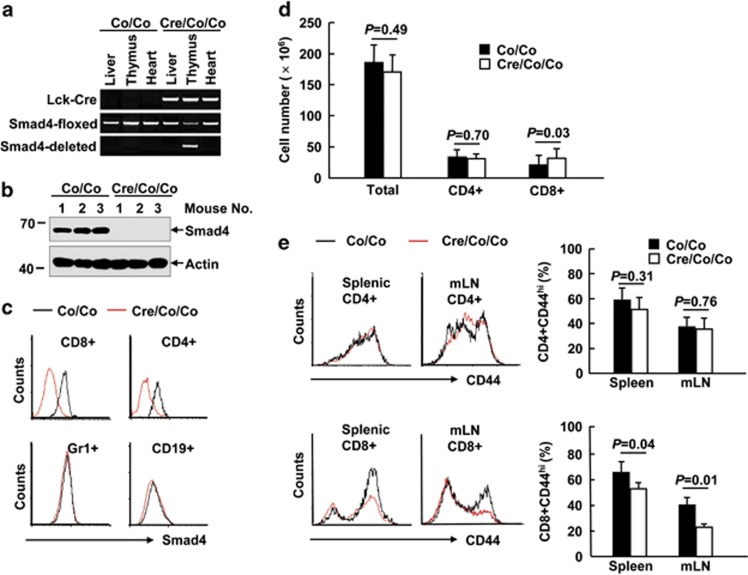
Eighteen-month-old *Smad4*^*co/co*^*;Lck-Cre* mice exhibit impaired CD44 expression in CD8^+^ T cells. (**a**) Genotyping of *Smad4*^*co/co*^*;Lck-Cre* mice (Cre/Co/Co) and control littermates (Co/Co). (**b**) The expression of Smad4 and actin in the thymocytes of 6- to 8-week-old *Smad4*^*co/co*^*;Lck-Cre* mice and control littermates. IB, immunoblotting. (**c**) Flow cytometry analysis of Smad4 expression in splenic CD4^+^ T, CD8^+^ T, Gr1^+^, and CD19^+^ B cells of 6- to 8-week-old *Smad4*^*co/co*^*;Lck-Cre* mice and control littermates. (**d**) The absolute numbers of total white cells, CD4^+^ T, and CD8^+^ T cells in the spleen, as revealed by white cell count and flow cytometry analysis, in 18-month-old *Smad4*^*co/co*^*;Lck-Cre* mice and control littermates (*n*=6 per group). (**e**) Flow cytometry analysis of CD44 expression in CD4^+^ and CD8^+^ T cells in the spleen and mesenteric lymph node (mLN) of 18-month-old *Smad4*^*co/co*^*;Lck-Cre* mice and control littermates (*n*=6 per group)

Specific inactivation of Smad4 in T cells was achieved by crossing mice homozygous for a *Smad4* conditional allele (*Smad4*^*co/co*^)^14, 15^ with mice expressing a transgene encoding Cre recombinase driven by the lymphocyte-specific protein tyrosine kinase (*Lck*) proximal promoter.^16^ Cre-mediated excision of exon 8 of the *Smad4* gene was detected by PCR (Figure 1a). Smad4 deficiency in thymocytes and splenic T cells was confirmed by immunoblotting and intracellular Smad4 staining (Figures 1b and c). However, levels of Smad4 were unaltered in other types of immune cells (Figure 1c). Compared to their littermate controls, *Smad4*^*co/co*^*;Lck-Cre* mice exhibited unchanged numbers of CD4^+^ splenic T cells as well as total splenocytes until 18-month old (Figure 1d). Furthermore, peripheral CD4^+^ T cells in 18-month-old *Smad4*^*co/co*^*;Lck-Cre* mice showed no aberrant CD44 expression (Figure 1e). However, Smad4 deficiency in T cells led to about 50% more CD8^+^ splenic T cells in 18-month-old mice (Figure 1d). Moreover, 18-month-old mice lacking Smad4 in T cells showed lower percentages of CD44^hi^CD8^+^ T cells both in the spleen and in the mesenteric lymph node (mLN; Figure 1e), suggesting that Smad4 deficiency in T cells might cause a defect in the activation/memory of CD8^+^ T cells.

### Unchanged antigen-specific CD8^+^ T-cell expansion in the absence of Smad4

**Figure 2:**
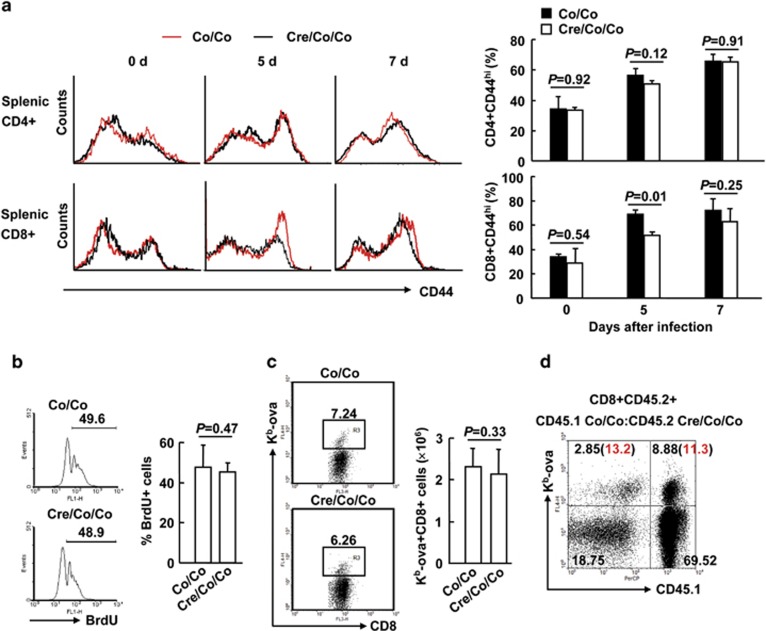
Unchanged antigen-specific CD8^+^ T-cell expansion in the absence of Smad4. (**a**–**c**) Six- to eight-week-old *Smad4*^*co/co*^*;Lck-Cre* mice and control littermates mice were infected with 5 × 10^3^ c.f.u. of LM-OVA (*n*=6 per group). (**a**) CD44 expression in CD4^+^ and CD8^+^ splenic T cells was analyzed by flow cytometry at days 0, 5, and 7 post infection. (**b**) Mice received 1 mg thymidine analog 5-bromo-2′-deoxyuridine (BrdU) in 0.1 ml PBS via i.p. injection at day 6 post infection; BrdU incorporation in CD8^+^ splenic T cells was analyzed by flow cytometry 24 h later. (**c**) The numbers of K^b^-ova^+^CD8^+^ splenic T cells were analyzed by flow cytometry at day 7 post infection. (**d**) Bone marrow chimeric mice reconstituted with a mix of *Smad4*^*co/co*^*;Lck-Cre* (CD45.2CD45.2) and *Smad4*^*co/co*^ (CD45.1CD45.2) cells were infected with 5 × 10^3^ c.f.u. of LM-OVA. Single-cell suspensions from the spleen were analyzed for the expression of CD45.1 and OVA specificity (K^b^-ova) at day 7 post infection. Representative plot of gated CD8^+^CD45.2^+^ T cells from the spleen is shown (*n*=3). The number in the bracket indicates the percentage of the K^b^-ova+ T cells in relation to CD8^+^ T cells of the same origin. Data shown in this figure are representative of at least three independent experiments

To explore the role of Smad4 in the activation of CD8^+^ T cells, we challenged 6- to 8-week-old *Smad4*^*co/co*^*;Lck-Cre* mice and their littermate controls with ovalbumin-modified *Listeria monocytogenes* (LM-OVA). At this age, basal CD44 expression in either CD4^+^ or CD8^+^ splenic T cells was unchanged in the absence of Smad4 (Figure 2a). LM-OVA infection led to CD44 upregulation in both CD4^+^ and CD8^+^ splenic T cells as the spleen is the primary site of infection (Figure 2a). Even though CD44 upregulation in CD8^+^ splenic T cells was partially impaired in *Smad4*^*co/co*^*;Lck-Cre* mice at day 5 post infection, it recovered at day 7 (Figure 2a). Moreover, the proliferation and expansion of CD8^+^ splenic T cells was unaffected in the absence of Smad4 at this time point (Figure 2b). As for OVA-antigen-specific T-cell responses, the frequencies and numbers of K^b^-ova^+^CD8^+^ splenic T cells were comparable between *Smad4*^*co/co*^*;Lck-Cre* mice and their littermate controls at day 7 post infection (Figure 2c). We also checked the proliferation of antigen-specific CD8^+^ splenic T cells at later time points. However, Smad4 deficiency did not affect the proliferation up to 14 days post infection (Supplementary Figure S1). To distinguish CD8^+^ T-cell-intrinsic or -extrinsic mechanisms underlying the unchanged antigen-specific T-cell expansion, we created mice with mixed bone marrow through transferring bone marrow cells from congenically marked *Smad4*^*co/co*^ (CD45.1CD45.2) and *Smad4*^*co/co*^*; Lck-Cre* (CD45.2CD45.2) mice into lethally irradiated CD45.1CD45.1 mice. After 8 weeks of bone marrow reconstitution, mice were infected with LM-OVA and the frequencies of K^b^-ova^+^CD8^+^ splenic T cells were assessed 7 days after infection. Flow cytometry analysis revealed that the frequencies of OVA-antigen-specific CD8^+^ T cells originating from the *Smad4*^*co/co*^*;Lck-Cre* bone marrow were similar to those of the *Smad4*^*co/co*^ counterparts in the same recipients (Figure 2d and Supplementary Figure S2). Thus, Smad4 plays a marginal role in the activation and proliferation of CD8^+^ T cells.

### Smad4 contributes to the cytotoxic function of CD8^+^ T cells

**Figure 3:**
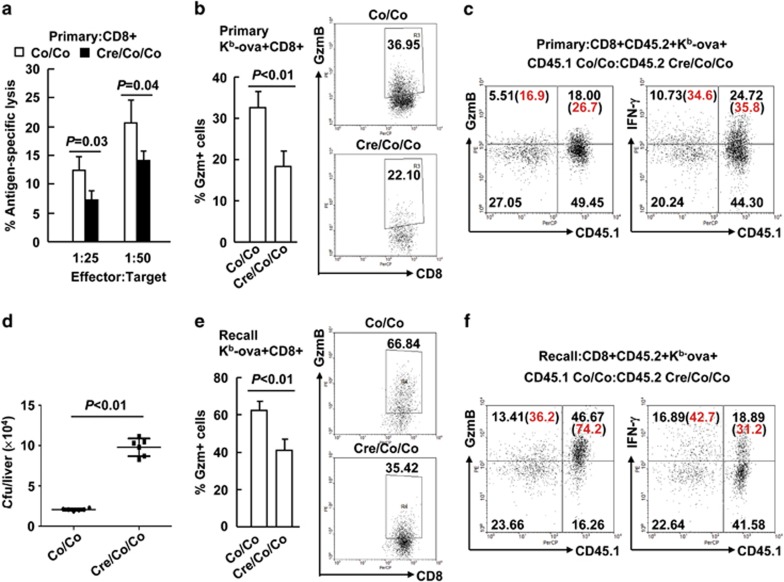
Smad4 is required for the cytotoxic function of CD8^+^ T cells. (**a** and **b**) Six- to eight-week-old *Smad4*^*co/co*^*;Lck-Cre* mice and their littermate controls were infected with 5 × 10^3^ c.f.u. of LM-OVA (*n*=6 per group). Single-cell suspensions from the spleen were prepared on day 7. (**a**) CD8^+^ cytotoxic activity was measured by specific killing of OVA peptide-loaded EL-4 cells. Peptide unloaded EL-4 cells were used as negative control. (**b**) GzmB expression in K^b^-ova^+^CD8^+^ splenic T cells upon restimulation with the SIINFEKL peptide (10 nM, 6 h) was analyzed by intracellular staining and flow cytometry. (**c**) Bone marrow chimeric mice were prepared and treated as described in Figure 2d. The expression of CD45.1 and GzmB in K^b^-ova^+^CD8^+^CD45.2^+^ splenic T cells at day 7 post infection upon OVA peptide restimulation was analyzed (*n*=3). (**d** and **e**) *Smad4*^*co/co*^*;Lck-Cre* mice and their littermates were rechallenged with 1 × 10^5^ c.f.u. of LM-OVA 35 days after primary infection (*n*=6 per group). (**d**) Bacterial burden in the liver was determined 2 days after the secondary infection. (**e**) GzmB expression in K^b^-ova^+^CD8^+^ splenic T cells upon OVA peptide restimulation was analyzed 5 days after the secondary infection. (**f**) Bone marrow chimeric mice were rechallenged with 1 × 10^5^ c.f.u. of LM-OVA 35 days after primary infection. The expression of CD45.1 and GzmB in K^b^-ova^+^CD8^+^CD45.2^+^ splenic T cells upon OVA peptide restimulation was analyzed 5 days after the secondary infection (*n*=3). Data shown in this figure are representative of at least three independent experiments

Next, whether Smad4 deficiency impacts on effector functions was explored 7 days after LM-OVA primary infection. Interestingly, Smad4-deficient CD8^+^ splenic T cells were partially impaired in their antigen-specific cytolytic activity (Figure 3a). Consistently, granzyme B (GzmB), the major mediator of CD8^+^ T-cell cytotoxicity, was partially absent from Smad4-deficient K^b^-ova^+^CD8^+^ splenic T cells upon restimulation with the SIINFEKL peptide (Figure 3b). To distinguish CD8^+^ T-cell-intrinsic or -extrinsic mechanisms underlying the defective GzmB expression, bone marrow chimeric mice stated above were analyzed at day 7 post infection of LM-OVA. Intracellular staining and flow cytometric analysis demonstrated that GzmB expression, but not interferon *γ* (IFN-*γ*) expression, in Smad4-deficient K^b^-ova^+^CD8^+^ splenic T cells was lower than that in competing Smad4-sufficient cells upon OVA peptide restimulation (Figure 3c and Supplementary Figure S3). Thus, Smad4 contributes to the cytotoxic function of CD8^+^ T cells in primary infection.

To explore whether Smad4 also controls the generation of cytotoxic effector cells from memory CD8^+^ T cells, we challenged *Smad4*^*co/co*^*;Lck-Cre* mice and their littermates with a higher dose of LM-OVA 35 days after primary infection. The bacterial burden in the liver, another primary site of infection, was examined 2 days after the secondary infection. *Smad4*^*co/co*^*;Lck-Cre* mice had about fivefold more *Listeria* colony-forming units (c.f.u.) than their littermate controls (Figure 3d), suggesting Smad4 deficiency led to defective generation of cytotoxic effector cells from memory CD8^+^ T cells. Consistently, Smad4 deficiency led to partially diminished GzmB expression in K^b^-ova^+^CD8^+^ splenic T cells upon OVA peptide restimulation 5 days after the secondary infection (Figure 3e). Despite that, Smad4-deficient antigen-specific CD8^+^ T cells showed unaffected expression of IFN-*γ* and TNF-*α* under the same conditions (Supplementary Figure S4). For bone marrow chimeric mice, intracellular staining and flow cytometric analysis demonstrated that Smad4-deficient K^b^-ova^+^CD8^+^ splenic T cells exhibited decreased GzmB expression, but statistically unaffected IFN-*γ* expression, upon OVA peptide restimulation 5 days after the secondary infection, as compared to competing Smad4-sufficient cells (Figure 3f and Supplementary Figure S5). Thus, Smad4 also contributes to the cytotoxic function of CD8^+^ T cells in recall responses.

### Smad4-deficient T cells show aberrant CD8^+^ T-cell differentiation

**Figure 4:**
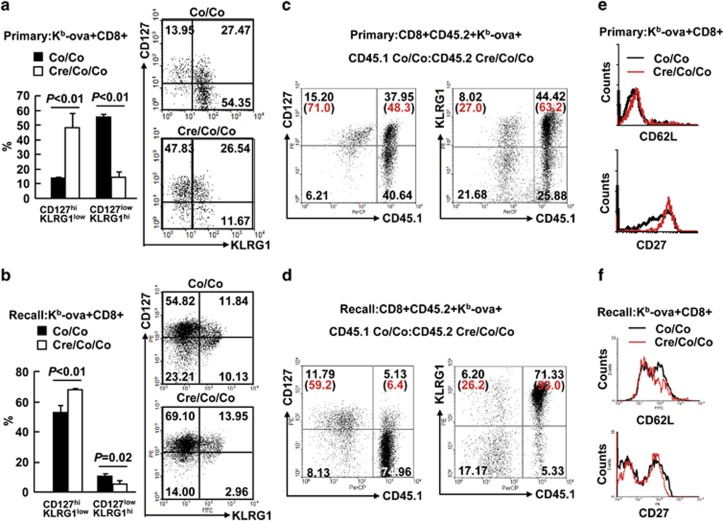
Smad4-deficient T cells show aberrant CD8^+^ T-cell differentiation. (**a**) The expression of CD127 and KLRG1 in K^b^-ova^+^CD8^+^ splenic T cells of *Smad4*^*co/co*^*;Lck-Cre* mice and their littermate controls on day 7 of LM-OVA primary infection (*n*=6 per group). (**b**) The expression of CD127 and KLRG1 in K^b^-ova^+^CD8^+^ splenic T cells of *Smad4*^*co/co*^*;Lck-Cre* mice and their littermate controls 5 days after the secondary infection (*n*=6 per group). (**c**) Bone marrow chimeric mice were prepared and treated as described in Figure 2d. The expression of CD45.1 and CD127 or KLRG1 in K^b^-ova^+^CD8^+^CD45.2^+^ splenic T cells at day 7 post infection was analyzed by flow cytometry (*n*=3). (**d**) Bone marrow chimeric mice were rechallenged with 1 × 10^5^ c.f.u. of LM-OVA 35 days after primary infection. The expression of CD45.1 and CD127 or KLRG1 in K^b^-ova^+^CD8^+^CD45.2^+^ splenic T cells 5 days after the secondary infection was analyzed by flow cytometry (*n*=3). (**e**) The expression of CD62L and CD27 in K^b^-ova^+^CD8^+^ splenic T cells of *Smad4*^*co/co*^*;Lck-Cre* mice and their littermate controls on day 7 of LM-OVA primary infection (*n*=6 per group). (**f**) The expression of CD62L and CD27 in K^b^-ova^+^CD8^+^ splenic T cells of *Smad4*^*co/co*^*;Lck-Cre* mice and their littermate controls 5 days after the secondary infection. Data shown in this figure are representative of at least three independent experiments

At the peak of the primary immune response to LM-OVA, about 55% Smad4-sufficient K^b^-ova^+^CD8^+^ splenic T cells expressed high amounts of KLRG1 and downregulated CD127 (Figure 4a), characteristics of SLEC. However, this number dropped to about 15% for their Smad4-deficient counterparts (Figure 4a). These data are consistent with our previous finding that Smad4 is required for the cytotoxic function of CD8^+^ T cells. However, about 15% K^b^-ova^+^CD8^+^ splenic T cells in *Smad4*^*co/co*^ mice were CD127^hi^KLRG1^low^, characteristics of memory-precursor effector cells (MPECs). This number increased to about 50% for their *Smad4*^*co/co*^*;Lck-Cre* counterparts (Figure 4a). To make it clear whether the Smad4-deficient CD8^+^ T cells maintain or re-express CD127 at high level at day 7 post infection of LM-OVA, we checked CD127 expression at earlier time points. We found that basal CD127 expression in CD8^+^ splenic T cells was unchanged in the absence of Smad4 (Supplementary Figure S6). At day 5 post infection of LM-OVA, a small portion of CD8^+^ splenic T cells in *Smad4*^*co/co*^ mice began to downregulate CD127. However, Smad4-deficient CD8^+^ splenic T cells showed no downregulation (Supplementary Figure S6). Thus, Smad4-deficient CD8^+^ T cells maintain high CD127 expression after LM-OVA infection. The failure to upregulate KLRG1 and the maintenance of high CD127 expression was a uniform feature of Smad4-deficient K^b^-ova^+^CD8^+^ splenic T cells as revealed by the fact that it occurred in both primary (Figure 4a) and recall responses (Figure 4b). Importantly, analysis of infected bone marrow chimeric mice at the same time points indicates that such a feature is CD8^+^ T-cell intrinsic (Figures 4c and d; Supplementary Figures S7 and S8). Despite the significant accumulation of the CD127^hi^KLRG1^low^ subset, flow cytometry analysis indicated that Smad4-deficient K^b^-ova^+^CD8^+^ splenic T cells exhibited enhanced expression of CD27, a cell-surface marker highly expressed in MPECs and has a pivotal role in the control of memory T-cell responses,^17^ only in primary (Figure 4e) but not in recall responses (Figure 4f). As for CD62L, another cell-surface marker essential for the control of memory T-cell responses,^18^ Smad4-deficient K^b^-ova^+^CD8^+^ splenic T cells failed to show enhanced expression of CD62L in both primary and recall responses (Figures 4e and f). These data suggest that Smad4 deficiency leads to aberrant CD8^+^ T-cell differentiation.

### Smad4-deficiency leads to defective memory

**Figure 5:**
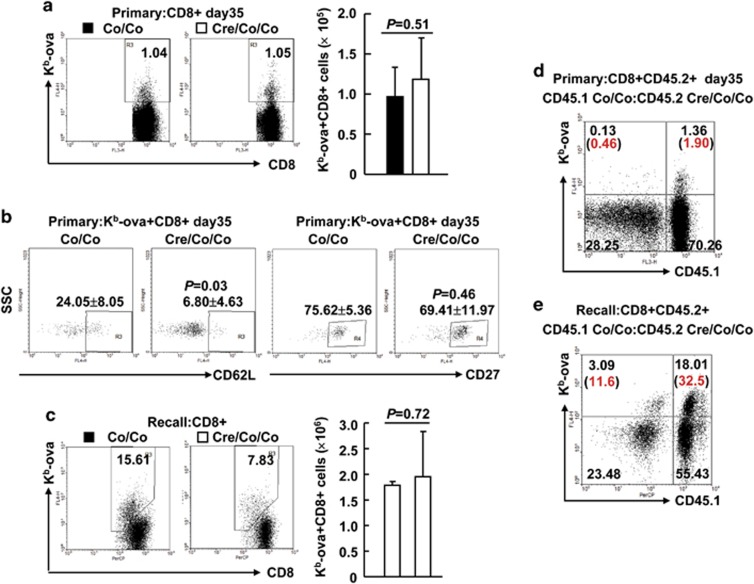
Smad4 deficiency leads to defective memory. (**a**) The percentages and numbers of K^b^-ova^+^CD8^+^ splenic T cells in *Smad4*^*co/co*^*;Lck-Cre* mice and their littermate controls on day 35 of LM-OVA primary infection (*n*=6 per group). (**b**) The expression of CD62L and CD27 in K^b^-ova^+^CD8^+^ splenic T cells of *Smad4*^*co/co*^*;Lck-Cre* mice and their littermate controls on day 35 of LM-OVA primary infection (*n*=6 per group). (**c**) The percentages and numbers of K^b^-ova^+^CD8^+^ splenic T cells in *Smad4*^*co/co*^*;Lck-Cre* mice and their littermate controls 5 days after the secondary infection (*n*=6 per group). (**d**) Bone marrow chimeric mice were prepared and treated as described in Figure 2d. The expression of CD45.1 and OVA specificity (K^b^-ova) in CD8^+^CD45.2^+^ splenic T cells at day 35 post infection was analyzed by flow cytometry (*n*=3). (**e**) Bone marrow chimeric mice were rechallenged with 1 × 10^5^ c.f.u. of LM-OVA 35 days after primary infection. The expression of CD45.1 and OVA specificity (K^b^-ova) in CD8^+^CD45.2^+^ splenic T cells 5 days after the secondary infection was analyzed by flow cytometry (*n*=3). Data shown in this figure are representative of at least three independent experiments

Smad4 deficiency partially affected effector T-cell differentiation and led to the accumulation of MPECs without the proper expression of certain cell-surface markers. To understand the effect of Smad4 loss on memory, we analyzed the proportion of OVA antigen-specific memory CD8^+^ T cells in Smad4-sufficient and -deficient CD8^+^ splenic T cells 35 days after primary infection. Neither the frequency nor the number of Smad4-deficient K^b^-ova^+^CD8^+^ splenic T cells was significantly different from that of control cells (Figure 5a). Smad4 deficiency showed no effect on CD27 expression, but led to diminished CD62L expression, in antigen-specific memory CD8^+^ T cells 35 days after primary infection (Figure 5b). In line with this, the frequency and the number of Smad4-deficient K^b^-ova^+^CD8^+^ splenic T cells were similar to those of control cells 5 days after the secondary infection (Figure 5c). Thus, the accumulation of MPECs did not result in enhanced memory. To further address this issue, infected bone marrow chimeric mice were analyzed at the same time points. We found that the frequencies of K^b^-ova^+^CD8^+^ splenic T cells originating from the *Smad4*^*co/co*^*;Lck-Cre* bone marrow were lower than those of *Smad4*^*co/co*^ counterparts in the same recipients 35 days after primary infection (Figure 5d and Supplementary Figure S9). Upon LM-OVA rechallenge, the ratios of Smad4-deficient K^b^-ova^+^CD8^+^ splenic T cells to competing Smad4-sufficient counterparts were further decreased (Figure 5e and Supplementary Figure S10). Taken together, these observations reveal a cell-intrinsic role for Smad4 in CD8^+^ T-cell memory.

### Smad4 contributes to the differentiation of central memory T cells by promoting the survival of MPECs

**Figure 6:**
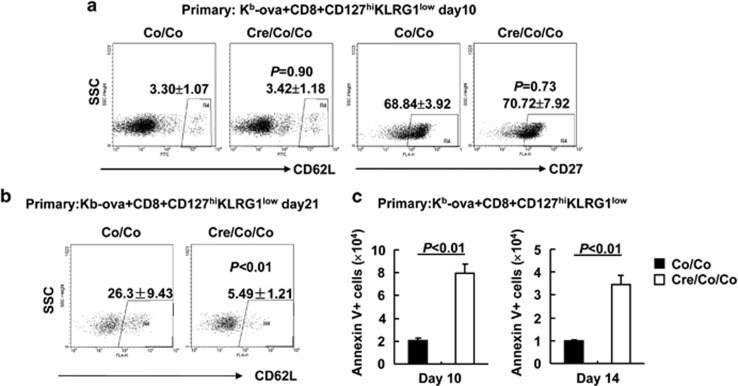
Smad4 contributes to the differentiation of central memory T cells by promoting the survival of MPECs. (**a** and **b**) The expression of CD62L and CD27 in K^b^-ova^+^CD8^+^CD127^hi^KLRG1^low^ splenic T cells of *Smad4*^*co/co*^*;Lck-Cre* mice and their littermate controls on day 10 (**a**) or day 21 (**b**) of LM-OVA primary infection (*n*=6 per group). (**c**) Apoptosis analysis of K^b^-ova^+^CD8^+^CD127^hi^KLRG1^low^ splenic T cells of *Smad4*^*co/co*^*;Lck-Cre* mice and their littermate controls on days 10 and 14 of LM-OVA primary infection (*n*=6 per group). Data shown in this figure are representative of at least three independent experiments

Next, we tried to explore why Smad4 deficiency leads to defective CD8^+^ T-cell memory despite of MPEC accumulation. We first checked the upregulation of central memory T-cell markers CD62L and CD27 on MPECs. At day 10 post infection of LM-OVA, Smad4-sufficient and -deficient K^b^-ova^+^CD8^+^ CD127^hi^KLRG1^low^ splenic T cells showed comparable percentages of CD27^hi^ cells (68.84±3.92% *versus* 70.72±7.92% Figure 6a). As CD62L was only marginally upregulated on MPECs under the same conditions (Figure 6a), we also checked CD62L expression on day 21 post infection. As expected, CD62L was significantly upregulated in K^b^-ova^+^CD8^+^CD127^hi^KLRG1^low^ splenic T cells at this time point (Figure 6b). However, its upregulation was impaired upon Smad4 deficiency (Figure 6b). These facts pushed us to examine the survival of MPECs of *Smad4*^*co/co*^*;Lck-Cre* mice and their littermate controls. As shown in Figure 6c, K^b^-ova^+^CD8^+^ CD127^hi^KLRG1^low^ splenic T cells showed impaired survival in the absence of Smad4. Thus, central memory T cells were generated normally in absence of Smad4 but the cells showed survival issue.

### Smad4 regulates the transcriptional program in antigen-specific T cells

**Figure 7:**
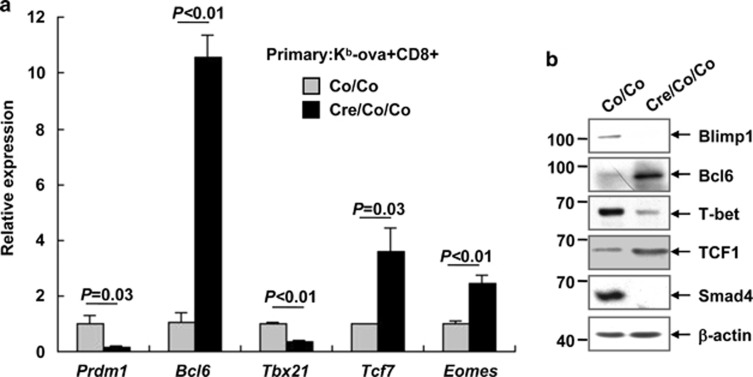
Smad4 regulates the transcriptional program in antigen-specific T cells. (**a** and **b**) K^b^-ova^+^CD8^+^ splenic T cells were sorted from *Smad4*^*co/co*^*;Lck-Cre* mice and their littermate controls on day 7 of LM-OVA primary infection. Three to four biologically independent samples with the same genotype were mixed together and the experiment was repeated three times (**a**). Cells were then subjected to quantitative RT-PCR for the indicated transcripts. Data are shown as the expression relative to that found in Smad4-sufficient K^b^-ova^+^CD8^+^ splenic T cells (arbitrarily set to 1). (**b**) Cell lysates were then prepared and subjected to immunoblotting analysis with the indicated antibodies

A small number of transcription factors, including Blimp1 (*Prdm1*), Bcl6, T-bet (*Tbx21*), *Tcf7*, and Eomes, are known to be important in the regulation of effector and memory CD8^+^ T-cell differentiation.^5, 6, 7, 8, 9, 10, 11^ Quantitative RT-PCR analysis revealed that Smad4-deficient K^b^-ova^+^CD8^+^ splenic T cells expressed reduced levels of *Prdm1* and *Tbx21* transcripts but enhanced levels of *Bcl6*, *Tcf7*, and *Eomes* transcripts 7 days after LM-OVA primary infection, as compared to Smad4-sufficient counterparts (Figure 7a). Immunoblotting analysis of antigen-specific CD8^+^ T cells purified at the same time point indicated the same tendency (Figure 7b). These data suggest that Smad4 is required to establish the transcriptional profile essential for proper CD8^+^ T-cell differentiation.

## Discussion

Smad4 is a key intracellular signaling effector for the TGF-*β* superfamily.^13^ TGF-*β* via Smad4 drives IL-10 expression in Th1 cells, IL-9 expression in Th9 cells, and IgA expression in B cells.^19, 20, 21^ It is reasonable to expect that mice with specific inactivation of Smad4 in T cells phenotypically resemble T-cell-specific TGF-*β*R-deficient mice and develop autoimmunity.^22, 23^ However, previous reports have demonstrated that Smad4 deficiency in T cells leads to proliferative epithelial lesions of the gastrointestinal tract, but not autoimmunity.^24, 25^ Consistently, people with germline mutations of Smad4 predispose to familial juvenile polyposis and gastrointestinal cancers but not some autoimmune diseases.^26, 27^ These facts suggest that Smad4 has TGF-*β*-independent functions in T cells. Indeed, a recent study has demonstrated that Smad4 contributes to T cells function during autoimmunity and anti-tumor immunity independent of TGF-*β*R signaling.^28^

CD8^+^ cytotoxic T cells play pivotal roles in autoimmunity and anti-tumor immunity as well as the clearance of intracellular pathogens. It has been revealed that Smad4 is essential for the development of central memory CD8^+^ T cells whereas TGF-*β* is dispensable.^29^ However, the underlying mechanisms by which Smad4 promotes the development of central memory CD8^+^ T cells remain unknown, and no data have been shown about the role of Smad4 in cytokine production of CD8^+^ T cells. Here, we confirm that Smad4 deficiency leads to reduced memory CD8^+^ T-cell responses. Further exploration revealed the accumulation of memory-precursor effector T cells in the absence of Smad4. Then, we have disclosed that defective survival leads to defective generation of central memory T cells in the absence of Smad4 due to aberrant transcriptional programs. Moreover, we show Smad4 deficiency partially impairs the production of GzmB, the major mediator of CD8^+^ T-cell cytotoxicity. These functions of Smad4 might contribute to its critical role in preventing tumor development.

In our model, Smad4 is deficient not only in CD8^+^ T cells but also in CD4^+^ T cells. Because of the essential effects of Smad4 in the control proper CD4^+^ T-cell differentiation,^19, 20, 28^ consequently changes in CD4^+^ T cell help or Treg function could alter the course of infection. Indeed, there are fewer Smad4-deficient K^b^-ova^+^CD8^+^ splenic T cells than competing Smad4-sufficient counterparts in chimeras 35 days post infection, while the frequencies and numbers of K^b^-ova^+^CD8^+^ splenic T cells are comparable between *Smad4*^*co/co*^*;Lck-Cre* mice and their littermate controls at the same time point. The changed microenvironment in *Smad4*^*co/co*^*;Lck-Cre* mice might contribute to the difference. Similarly, the changed microenvironment in *Smad4*^*co/co*^*;Lck-Cre* mice might also blur the bacteria burdens measured 5 days after the secondary infection. The data obtained from chimeras are more convincing. This approach ensures that Smad4-deficient and Smad4-sufficient CD8^+^ T cells are exposed to identical concentrations of antigen and inflammation during infection. Therefore, whether defective generation of central memory T cells associated with survival issue in *Smad4*^*co/co*^*;Lck-Cre* mice is cell-intrinsic defects should be further confirmed with chimeras in the future. With chimeras, we have observed both defective memory and impaired production of GzmB in the absence of Smad4, consistent with the data obtained from *Smad4*^*co/co*^*;Lck-Cre* mice despite of minor differences. The use of different mixed bone marrow chimeras and analysis at different time points after infection would make the conclusions more convincing.

Improved understanding of the generation of effector and memory CD8^+^ T cells during the immune response could be used to augment vaccine-induced immunity. Our findings suggest manipulating Smad4 functions might facilitate the control of infection, autoimmunity, and cancer. The TGF-*β*-independent functions of Smad4 in CD8^+^ T-cell differentiation may be downstream of signaling, which begins with the receptor for bone morphogenic protein or another unidentified cytokine. The source of such a cytokine may be T cells and other types of cells in the microenvironment. Enhancing or suppressing the production of such a cytokine might be an easy way to control infection, autoimmunity, and cancer.

## Materials and Methods

### Mice

Mice homozygous for a *Smad4* conditional allele (*Smad4*^*co/co*^) were previously described^14, 15^ and were all backcrossed to the C57BL/6 background. Mice expressing a transgene encoding Cre recombinase driven by *Lck* proximal promoter were purchased from the Jackson Laboratory (Bar Harbor, ME, USA). Specific inactivation of Smad4 in T cells was achieved by crossing *Smad4*^*co/co*^ mice with *Lck*-*Cre* mice. All mice were maintained under specific pathogen-free conditions, and animals were handled in accordance with institutional guidelines. Genotyping was performed as described previously.^30^

### *Listeria monocytogenes* infection

For the study of primary immune response, mice were intravenously infected with 5 × 10^3^ c.f.u. of LM-OVA. For the analysis of secondary immune response, mice were rechallenged with 1 × 10^5^ c.f.u. of LM-OVA 35 days after primary infection.

### BrdU incorporation

Staining of BrdU incorporation followed the BrdU Flow kit protocol (Becton Dickinson, San Jose, CA, USA). Briefly, cells were dehydrated in an alcohol solution, fixed and permeabilized in 1% paraformaldehyde/0.01% Tween 20, treated with 50 U/ml DNase, and then stained with 10 *μ*l of FITC-conjugated anti-BrdU (Becton Dickinson).

### Colony-forming unit assay

Single-cell suspensions were made from livers in PBS containing 0.01% Triton X-100 (Sigma, St. Louis, MO, USA). The supernatants were inoculated on brain-heart-infusion agar plates and incubated for 24 h at 37 °C. Bacterial colonies were counted.

### Flow cytometry

Fluorescent-dye-labeled antibodies against cell-surface markers CD4, CD8, CD44, CD62L, Gr1, CD19, CD45.1, CD45.2, CD127, CD27, and KLRG1 were purchased from eBioscience (San Diego, CA, USA). FITC- or PE-conjugated antibodies against cytokines were from BioLegend (San Diego, CA, USA). APC- or PE-conjugated K^b^-ova+ tetramer was obtained from QuantoBio (Beijing, China). Splenic cells were depleted of erythrocytes by hypotonic lysis. The cells were washed with FACS washing buffer (2% FBS, 0.1% NaN_3_ in PBS) twice and were then incubated with fluorescence-conjugated antibodies against cell-surface molecules for 30 min on ice in the presence of 2.4G2 mAb to block Fc*γ*R binding. Isotype antibodies were included as negative controls. For intracellular cytokine staining, single-cell suspensions were stimulated with 10 nM SIINFEKL peptide in the presence of Brefeldin A solution (eBioscience) for 6 h at 37 °C. After stimulation, cells were stained with fluorescence-conjugated antibodies against cell-surface markers, fixed, and permeabilized using a fixation/permeabilization kit (eBioscience) and stained with fluorescence-conjugated specific antibodies against Smad4 (Santa Cruz, Santa Cruz, CA, USA), IFN-*γ*, TNF-*α*, and GzmB in accordance with the manufacturer's instructions. Flow cytometry was performed using a Becton Dickinson FACSCalibur machine.

### Antibodies and immunoblotting

Antibodies against Blimp1 (9115 S, encoding by *Prdm1*) and TCF-1 (2203S, encoding by *Tcf7*) were purchased from Cell Signaling (Beverly, MA, USA). Antibodies against Smad4 (sc-7966) and *β*-actin (sc-8432) were obtained from Santa Cruz. Antibodies against Bcl6 (648301) and T-bet (644801, encoding by *Tbx21*) were obtained from BioLegend. Total protein extracts were prepared and dissolved in SDS sample buffer. Protein extracts were separated on 10% SDS-PAGE gels and transferred to polyvinylidene difluoride membranes (Millipore, Billerica, MA, USA). The membranes were probed with antibodies and visualized with an electrochemiluminescence kit (Amersham, Uppsala, Sweden).

### Generation of bone marrow chimeras

Bone marrow cells were depleted of T cells and antigen-presenting cells by complement-mediated cell lysis. *Smad4*^*co/co*^ and *Smad4*^*co/co*^*;Lck-Cre* bone marrow cells (2 × 10^6^) were cotransferred into lethally irradiated recipients (1200 rads). The congenic markers CD45.1 and CD45.2 were used for distinguishing cells from different donors and recipients.

### Cytotoxicity assays

CD8^+^ cytotoxic function was analyzed using flow cytometry as described previously.^31, 32^ Briefly, single-cell suspensions from the spleen were prepared on day 7 of LM-OVA infection. Purified CD8^+^ T cells were used as effector cells. EL-4 cells were pulsed with 2 *μ*M OVA_257–264_ (SIINFEKL) peptide at 37 °C for 1 h. Peptide-pulsed EL-4 cells were used as target cells with unpulsed EL-4 cells as negative control. Target cells (2 × 10^4^ per well) were cocultured with effector cells in 96 U-bottom plates at 25 : 1 and 50 : 1. After incubation for 6 h at 37 °C, cells were collected and stained with FITC-conjugated annexin V, PERCP-conjugated anti-CD8 and propidium iodide (PI), followed by flow cytometry. Specific lysis (%) was calculated as follows: CD8^−^ cells minus CD8-annexin V-PI^−^ cells/CD8^−^ cells × 100. Spontaneous apoptosis was <10% for all experiments.

### Apoptosis analysis

Single-cell suspensions from the spleen were prepared on days 10 and 14 of LM-OVA infection. Purified CD8^+^ T cells were stained with anti-K^b^-ova-PE, anti-KLGR1-FITC, anti-CD127-PERCP, and annexin V-APC resuspended in 300 *μ*l binding buffer containing calcium ion. Apoptosis was assessed by flow cytometric analysis of annexin-V staining in K^b^-ova^+^CD8^+^ CD127^hi^KLRG1^low^ splenic T cells.

### Quantitative RT-PCR

RNA was extracted by using TRIzol reagent (Invitrogen, Carlsbad, CA, USA). First-strand synthesis was performed with oligo-dT primers, and reverse transcription was performed with M-MLV reverse transcriptase. Quantitative real-time PCR was performed using SYBR Green reagent (TOYOBO, Tokyo, Japan) in a real-time PCR machine Realplex 2 (Eppendorf, Hamberg, Germany). Reactions were performed three times independently, and GAPDH values were used to normalize gene expression. Primers for murine *Prdm1*, *Bcl6*, and *Tbx21*;^11^ primers for murine *Eomes*;^10^ and primers for murine *Tcf7*^33^ have been reported previously. Primers for GAPDH were 5′-ggcaaattcaacggcacagt-3′ (forward) and 5′-agatggtgatgggcttccc-3′ (reverse).

### Statistics

Results are shown as mean±S.D. Differences were considered significant with a *P*-value <0.05 using Student's *t*-test (paired or unpaired) and one-way analysis of variance (ANOVA).

## Abbreviations

KLRG1: killer cell lectin-like receptor G1
SLEC: short-lived effector T cells
Eomes: eomesodermin
Bcl6: B-cell CLL/lymphoma 6
Tcf7: T-cell factor 7
Blimp1: B lymphocyte-induced maturation protein 1
mLN: mesenteric lymph node
GzmB: granzyme B
MPECs: memory-precursor effector cells
*Lck*: lymphocyte-specific protein tyrosine kinase
c.f.u.: colony-forming units
LM-OVA: ovalbumin*-*modified *Listeria monocytogenes*
