# Supplementary material for: Smad4 represses the generation of memory-precursor effector T cells but is required for the differentiation of central memory T cells
Source: Cell Death Dis. 2015 Nov 19;6(11):e1984–. doi: 10.1038/cddis.2015.337 (PMC4670941; doi:10.1038/cddis.2015.337)
Supplement: Supplementary Information [file cddis2015337x1.pdf]

Supplementary Figures

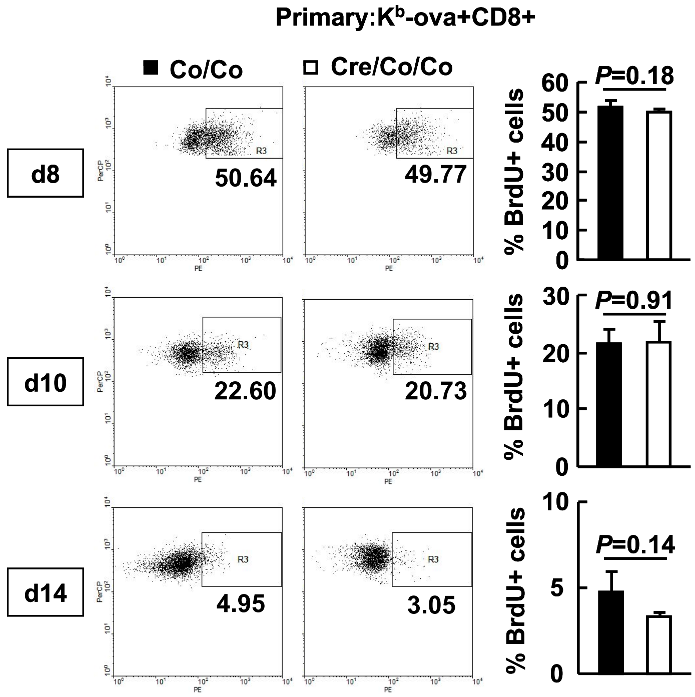

Figure S1 Analysis of the proliferation of antigen-specific CD8+ splenic T cells with BrdU incorporation 8, 10, 14 days postinfection.

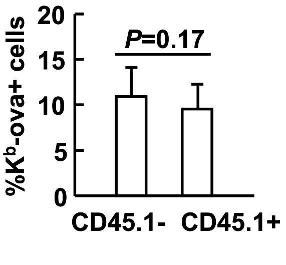

Figure S2 Statistical data of Figure 2d.

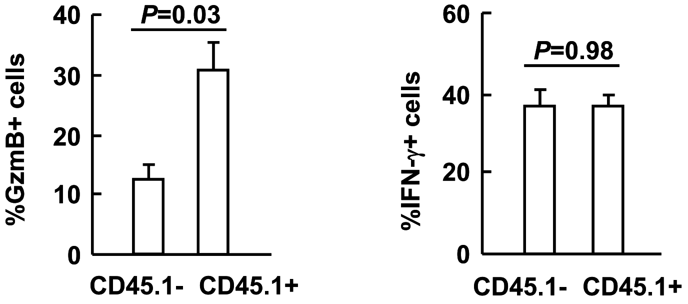

Figure S3 Statistical data of Figure 3c.

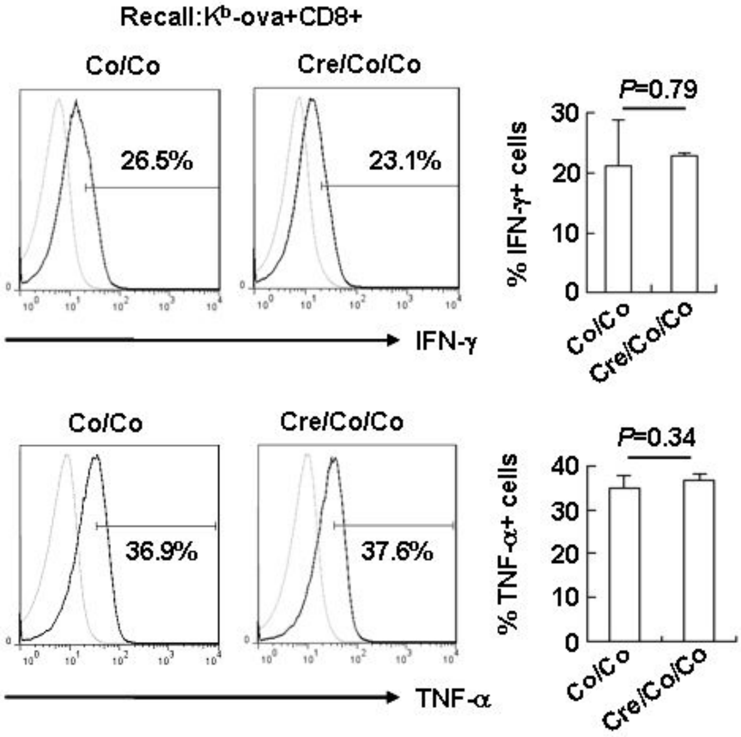

Figure S4 The expression of IFN-γ and TNF-α in K^b-ova+CD8+ splenic T cells upon OVA peptide restimulation was analyzed 5 days after the secondary infection.

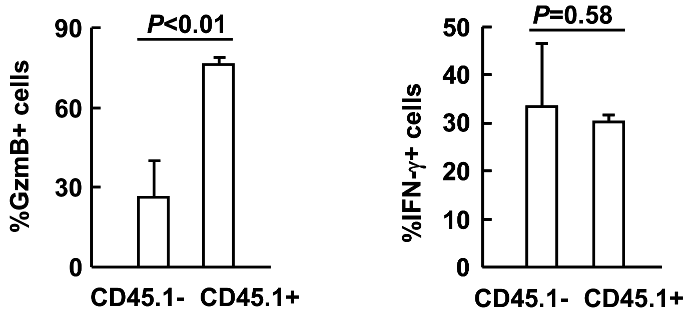

Figure S5 Statistical data of Figure 3f.

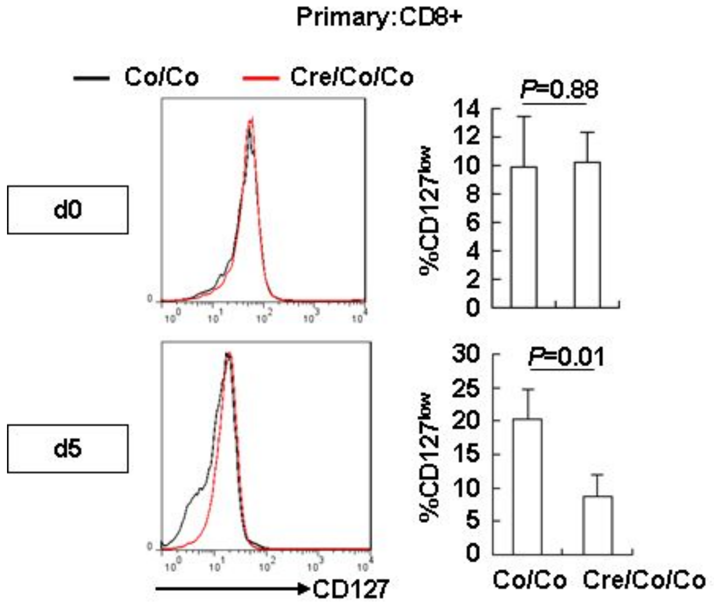

Figure S6 CD127 expression in CD8+ splenic T cells at day 0 and day 5 postinfection.

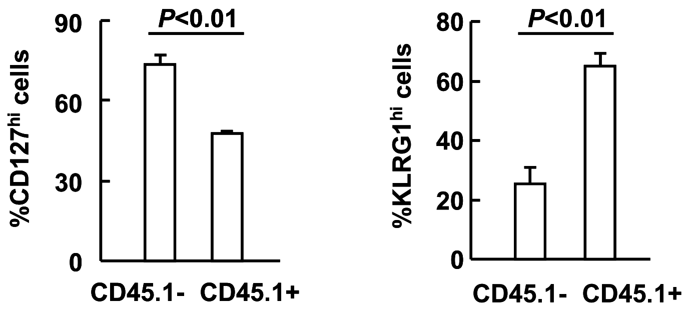

Figure S7 Statistical data of Figure 4c.

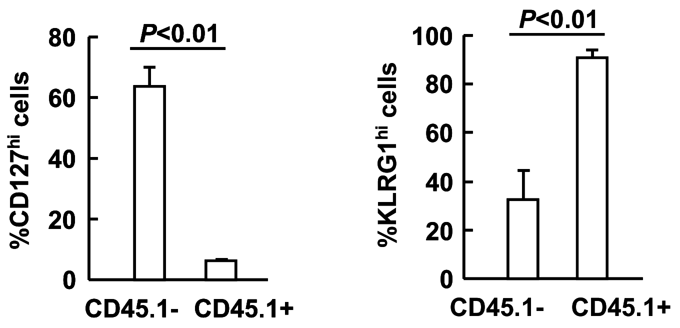

Figure S8 Statistical data of Figure 4d.

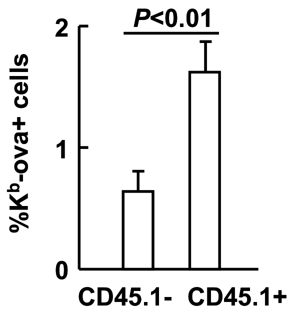

Figure S9 Statistical data of Figure 5d.

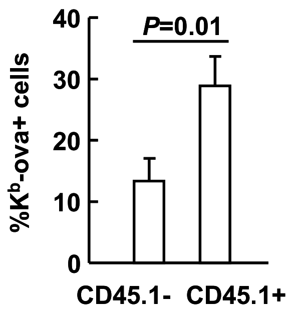

Figure S10 Statistical data of Figure 5e.
